# Effect of propranolol and clonidine after severe traumatic brain injury: a pilot randomized clinical trial

**DOI:** 10.1186/s13054-023-04479-6

**Published:** 2023-06-09

**Authors:** Mina F. Nordness, Amelia W. Maiga, Laura D. Wilson, Tatsuki Koyama, Erika L. Rivera, Shayan Rakhit, Michael de Riesthal, Cari L. Motuzas, Madison R. Cook, Deepak K. Gupta, James C. Jackson, Shawniqua Williams Roberson, William J. Meurer, Roger J. Lewis, Geoffrey T. Manley, Pratik P. Pandharipande, Mayur B. Patel

**Affiliations:** 1 Critical Illness, Brain Dysfunction, and Survivorship Center, Vanderbilt University Medical Center (VUMC), Suite 450, 4th Floor, 2525 West End Avenue, Nashville, TN 37203 USA; 2 Section of Surgical Sciences, Division of Acute Care Surgery, Department of Surgery, VUMC, 1211 21st Avenue South, Medical Arts Building, Suite 404, Nashville, TN 37212 USA; 3 Department of Hearing & Speech Sciences, VUMC, 1215 21st Avenue South, Medical Center East, Room 8310, Nashville, TN 37232 USA; 4 College of Health Sciences & Communication Sciences and Disorders at the University of Tulsa, 800 S Tucker Drive, Tulsa, OK 74104 USA; 5 Department of Biostatistics, VUMC, Room 11133B, 2525 West End Avenue, Nashville, TN 37203 USA; 6 Department of Radiology and Radiological Sciences, VUMC, Medical Center North, 1161 21st Avenue South, Nashville, TN 37232 USA; 7 Meharry Medical College, 1005 Dr. DB Todd Jr Blvd, Nashville, TN 37208 USA; 8 Division of Cardiovascular Medicine, Vanderbilt Translational and Clinical Cardiovascular Research Center, VUMC, 2525 West End, Suite 300-A, Nashville, TN 37203 USA; 9 University of Michigan Emergency Medicine, 1500 East Medical Center Drive, Ann Arbor, MI 48109 USA; 10 Department of Emergency Medicine, Harbor-University of California Los Angeles, 1000 W Carson St, Torrance, CA 90502 USA; 11 Department of Neurological Surgery, University of California San Francisco, 505 Parnassus Ave, Room M779, Box 0112, San Francisco, CA 94143 USA; 12 Center for Health Services Research, Institute for Medicine and Public Health, VUMC, 2525 West End Avenue, Nashville, TN 37203 USA; 13 Division of Anesthesiology Critical Care Medicine, Department of Anesthesiology, VUMC, 1211 Medical Center Drive, Nashville, TN 37232 USA; 14 Vanderbilt Brain Institute, VUMC, 7203 Medical Research Building III, 465 21st Avenue South, Nashville, TN USA; 15 Geriatric Research, Education and Clinical Center (GRECC), Nashville Veterans Affairs Medical Center, Tennessee Valley Healthcare System, 1310 24th Avenue South, Nashville, TN 37212 USA; 16 Surgical Services at the Nashville Veterans Affairs Medical Center, Tennessee Valley Healthcare System, 1310 24th Avenue South, Nashville, TN 37212 USA

**Keywords:** Traumatic brain injury, Critical care, Adrenergic blockade, Paroxysmal sympathetic hyperactivity

## Abstract

**Objective:**

To evaluate the safety, feasibility, and efficacy of combined adrenergic blockade with propranolol and clonidine in patients with severe traumatic brain injury (TBI).

**Background:**

Administration of adrenergic blockade after severe TBI is common. To date, no prospective trial has rigorously evaluated this common therapy for benefit.

**Methods:**

This phase II, single-center, double-blinded, pilot randomized placebo-controlled trial included patients aged 16–64 years with severe TBI (intracranial hemorrhage and Glasgow Coma Scale score ≤ 8) within 24 h of ICU admission. Patients received propranolol and clonidine or double placebo for 7 days. The primary outcome was ventilator-free days (VFDs) at 28 days. Secondary outcomes included catecholamine levels, hospital length of stay, mortality, and long-term functional status. A planned futility assessment was performed mid-study.

**Results:**

Dose compliance was 99%, blinding was intact, and no open-label agents were used. No treatment patient experienced dysrhythmia, myocardial infarction, or cardiac arrest. The study was stopped for futility after enrolling 47 patients (26 placebo, 21 treatment), per a priori stopping rules. There was no significant difference in VFDs between treatment and control groups [0.3 days, 95% CI (− 5.4, 5.8), *p* = 1.0]. Other than improvement of features related to sympathetic hyperactivity (mean difference in Clinical Features Scale (CFS) 1.7 points, CI (0.4, 2.9), *p* = 0.012), there were no between-group differences in the secondary outcomes.

**Conclusion:**

Despite the safety and feasibility of adrenergic blockade with propranolol and clonidine after severe TBI, the intervention did not alter the VFD outcome. Given the widespread use of these agents in TBI care, a multi-center investigation is warranted to determine whether adrenergic blockade is of therapeutic benefit in patients with severe TBI.

*Trial Registration Number* NCT01322048.

## Background

Severe traumatic brain injury (TBI) is associated with increased intracranial pressure, activation of the sympathetic nervous system and catecholamine response, and major morbidity and mortality [1–6]. This increased catecholamine response is predictive of length of stay, mechanical ventilation, neurologic outcome, and mortality [7]. Prior retrospective studies [8–12], including two from our group [13, 14], link adrenergic blockade to survival after severe TBI, possibly mediated by dampened sympathetic hyperactivity.

These findings have resulted in an increase in *β*-blocker use in our institution to greater than 40% in young, severe TBI patients [14]. *β*-blockade is just one pharmacologic strategy to reduce sympathetic hyperactivity; centrally acting *α*_2_-agonists also serve as sympatholytic agents [15–17]. The prototypical centrally acting *α*_2_-agonist, clonidine, decreases plasma catecholamines and improves outcomes in a rat model of incomplete cerebral ischemia [18]. Clonidine decreases plasma catecholamines and cerebral vasoconstriction without altering cerebral blood flow in patients with severe TBI [19, 20].

Despite available cohort and open-label data, rigorous blinded prospective trial evidence remains lacking on the feasibility, safety, and efficacy of *β*-blockers and *α*_2_-agonists after severe TBI [21–24]. A 2017 meta-analysis specifically highlighted the poor quality of the literature on this question and the need for high-quality prospective trials investigating beta blockade in TBI patients [20]. We conducted a single-center, double-blind, pilot randomized placebo-controlled trial to test the hypothesis that sympathetic blockade with propranolol and clonidine improves clinical outcomes in severe TBI patients by increasing ventilator-free days, defined as days alive and not on the ventilator, in addition to demonstrating the safety and feasibility of such a trial.

## Methods

### Trial design

We conducted a phase II, single-center, randomized, double-blinded, placebo-controlled pilot trial to test whether sympathetic blockade with propranolol and clonidine within 48 h of severe TBI improves clinical outcomes. Our protocol was approved by our Institutional Review Board and registered (NCT01322048). Enrollment took place between August 2011 through January 2015. One group received centrally acting sympatholytic drugs, propranolol and clonidine, and the other group received double placebo with identical routes of administration. To maintain group balance for this small trial, patients were assigned to treatment groups using a Bayesian weighted 1:1 adaptive co-variate randomization scheme with a random element based on neuroradiologist-rated computed tomography (CT) Marshall Class and age (double-weighted). At 50% accrual, two-stage stopping rules were applied on efficacy with Type I error of 3% and futility with conditional power < 50%.

### Participants

Inclusion criteria were severe TBI defined as Glasgow Coma Score (GCS) less than 8 with hemorrhage on head CT in patients between the ages of 16 and 64. Participants may or may not have had associated extracranial injuries. By definition, patients with TBI without hemorrhage on CT (i.e., diffuse axonal injury visible on magnetic resonance imaging [MRI] only) were not included. Exclusion criteria included pre-existing heart disease, study drug contraindication, penetrating TBI, pre-injury brain dysfunction, spinal cord injury, current *β*-blocker or *α*_2-_agonist use, and impending herniation, craniotomy, or death. Prisoners, pregnant women, and those unable to complete all follow-up visits were also excluded.

**Fig. 1:**
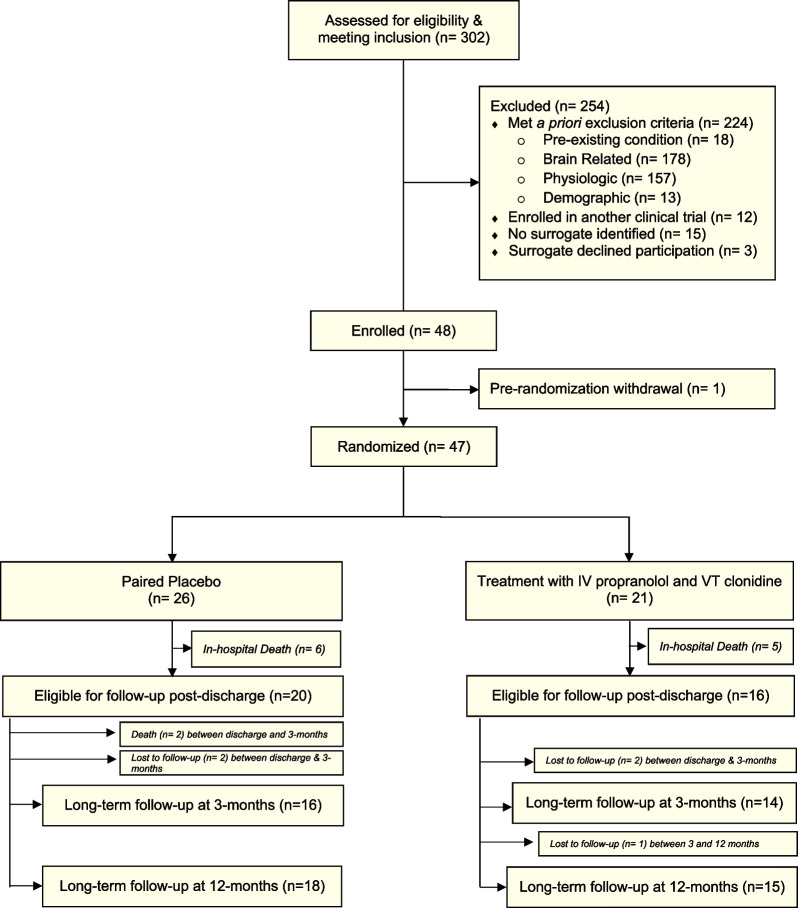
Screening, enrollment, and randomization for propranolol and clonidine versus double placebo after severe traumatic brain injury

Surrogates of patients who met inclusion criteria and no exclusion criteria were approached for enrollment, and informed consent was obtained from the designated surrogate before any study procedures were performed. Participants were re-consented if they regained capacity. Patients without surrogates were not enrolled. Potential participants had to be screened within 24 h of injury, with study drug initiation within 48 h of injury. Figure 1 shows the study CONSORT diagram [25].

### Intervention

Patients were administered either both propranolol and clonidine (treatment arm), or placebo. All patients and study personnel, except for the pharmacist and biostatistician, were blinded to treatment assignment. Treatment (or placebo) delivery started within 48 h of injury but after plasma and 24-h urine catecholamine measurements were obtained. Both drugs (or double placebos) were administered at staggered times for 7 days with drug holds for hemodynamic parameters [26]. Propranolol was administered intravenously at a dose of 1 mg every 6 h, and doses were held for heart rate less than 60 bpm, mean arterial pressure less than 60 mmHg, and/or cerebral perfusion pressure less than 60 mmHg. Placebo was intravenous administration of sterile 0.9% normal saline. Clonidine was administered at a dose of 0.1 mg per tube every 12 h and was held for mean arterial pressure < 60 mmHg and/or cerebral perfusion pressure < 60 mmHg. Placebo was per tube administration of microcrystalline cellulose compounded by the investigational drug service. If hemodynamic parameters were met while a patient was on vasopressors, drug delivery still occurred. We documented compliance with treatment delivery and any reasons for missed administrations.

Clinicians were allowed to use *β*-blockers at any point if there was a myocardial infarction or need for heart rate control refractory to calcium channel blockers and anti-arrhythmic medications. Dexmedetomidine, a prototypical *α*_2_-agonist, was allowed if standard sedation regimens, such as propofol, lorazepam, and midazolam, failed to achieve adequate sedation. In the case of vasopressors, phenylephrine and vasopressin were preferred agents, with additional support from inotropes dobutamine and milrinone. Norepinephrine, epinephrine, and dopamine agents were avoided unless increased cardiac output was needed or further systemic vascular resistance was required beyond that produced by preferred vasopressors. We tracked the use of non-study *β*-blockers and *α*_2_-agonists. Ventilators were weaned utilizing clinical judgment by an interdisciplinary team of respiratory therapists, critical care fellows, and attending physicians, who conducted twice-daily simultaneous spontaneous awakening and breathing trials, a practice developed at our institution [27].

### Safety monitoring

This phase II study focused on safety and efficacy. Safety parameters collected throughout the study included cardiac complications such as dysrhythmia (e.g., symptomatic bradycardia), myocardial infarction, cardiac arrest, and neuro-worsening. All serious, unexpected, and study-related adverse events were reported in a blinded fashion to the Data Safety Monitoring Board within 24 h.

### Efficacy outcomes

The primary outcome was ventilator-free days (VFD), defined as days alive and not requiring mechanical ventilation during a 28-day period, chosen to avoid the competing risk of mortality [28, 29]. Secondary outcomes included change in plasma and urine catecholamine levels following treatment (measured at enrollment/pre-randomization and study day 8), coma-free days, ICU length of stay (LOS), hospital LOS, in-hospital agitation, in-hospital mortality, and functional status at discharge. Arousal and agitation were measured twice daily with both the Richmond Agitation-Sedation Score (RASS) [30], Glasgow Coma Scale (GCS), and the Agitation Behavior Scale (ABS) for TBI until hospital discharge. Paroxysmal sympathetic hyperactivity (PSH) was measured using the validated Clinical Features Scale (CFS) [31]. At the time the study protocol was developed in 2011, the Paroxysmal Sympathetic Hyperactivity-Assessment Measure (PSH-AM) [31], which contains the CFS and the Diagnostic Likelihood Tool (DLT), had not yet been developed. However, at the time, the components of the CFS were well known as important diagnostic criteria for PSH [32] and thus, were prospectively collected. As the PSH-AM, including the CFS, was published during the conduct of the trial, these components of the CFS, identified a priori were used to calculate the CFS.

Additional secondary outcomes included neuropsychological and quality of life metrics obtained at in-person long-term follow-up. Study personnel obtained a baseline neuropsychological evaluation at hospital discharge using the Rancho Los Amigos level of cognitive functioning scale [33]. Follow-up visits at 3 and 12 months included neuropsychological testing for global executive function and processing speed using the Trail Making Test Part B [34]. Quality of life and functional status were assessed with the Quality of Life after Brain Injury (QOLIBRI) [35] and Extended Glasgow Outcome Scale (GOSE) [36]. The Social Security Death Index was monitored monthly to assess out-of-hospital mortality.

### Sample size calculation

Our sample size was calculated based on our primary outcome of ventilator-free days (VFD) after adrenergic blockade in severe TBI patients. As this was a pilot study that is the first of its kind, we utilized a clinically relevant difference between the experimental and control mean VFD of 2 days, with a standard deviation of 3 days; we calculated that 48 experimental and 48 control subjects were required to achieve 90% power, plus accounting for 4 patient withdrawals. A planned interim analysis (described in detail in our “Interim Analysis Plan”) accounts for the uncertainty in the true difference in VFD [37]. The Type I error rate was set at 5%. With independent biostatistical input, this calculation was performed using PS, Power and Sample Size Calculations program [38]. The primary outcome was VFD with a planned enrollment of 100 subjects. At 50% accrual, the two-stage stopping rules were based on efficacy with Type I error of 3% and futility with conditional power < 50%, utilizing an alpha spending approach to account for multiple tests [37]. This a priori planned futility assessment was performed mid-way through participant accrual, while maintaining the blind, to determine either overwhelming evidence of efficacy or futility.

### Randomization

In order to maintain group balance, patients were assigned to treatment groups using a Bayesian weighted adaptive co-variate randomization scheme with a random element based on CT Marshall Class and age, with age weighted twice the CT Marshall Class [39, 40]. This allocation scheme allowed a dominant element of randomness while achieving balance between the two groups. Randomization was performed by investigational pharmacists using a password-protected computer program.

### Blinding

Apart from investigational pharmacy and study biostatisticians, all clinical and research personnel were blinded to each patient’s treatment group. Placebo, both injections and tablets, were indistinguishable from active treatments when sent from the investigational pharmacy.

### Statistical methods

Data were analyzed with an intention-to-treat approach. Continuous data were summarized using means and standard deviations, while categorical data were summarized using frequencies and proportions. Differences between the treatment groups on continuous variables were assessed using Wilcoxon rank-sum tests, and for categorical variables, difference in proportions was estimated with the Pearson method. In-hospital mortality was compared using Fisher’s exact test. Survival proportions were estimated with Kaplan–Meier method, and log-rank test was used to assess the difference in survival function. A significance level of 0.05 was used to indicate statistical significance. Secondary neurocognitive outcomes were assessed in survivors who successfully completed long-term follow-up. All analyses were completed using R statistical software version 3.4.

## Results

Over a four-year period, we screened 302 consecutive patients with severe TBI admitted to our center. Enrollment was stopped at 50% accrual at 48 patients as the futility stopping rule determined that, even at its accrual goal of 96, the trial would not achieve conditional power to detect a significant difference in the primary outcome. A single patient was withdrawn from the study by their surrogate prior to randomization, leaving 47 patients randomized to placebo or treatment. Figure 1 shows our screening, enrollment, randomization, and follow-up. A total of 26 patients were allocated to the placebo group and 21 to the treatment group. A total of 11 patients died in-hospital, 6 in the placebo group and 5 in the treatment group, leaving a total of 20 and 16 in the placebo and treatment groups, respectively, for follow-up. Two patients in the placebo group died before their 3-month follow-up. Two patients in the treatment group completed the 3-month assessment but were lost to follow-up at the 12-month assessment. All other patients completed the study and were observed for their entire hospitalization or until study day 28. Among 47 randomized, the age, sex, race, injury and imaging severity, and organ failure score are listed in Table 1. Total hospital doses of fentanyl and propofol were comparable between groups. In terms of sedatives other than fentanyl or propofol, two patients received phenobarbital (both < 60 mg per day), and one patient received midazolam (< 40 mg per day).

**Table 1:** Clinical data: baseline characteristics and outcomes of propranolol and clonidine (treatment) versus double placebo after severe traumatic brain injury

Clinical data	Placebo (*n* = 26)	Treatment (*n* = 21)	*P* value
Baseline characteristics			
Age, in median (IQR)	28 (19–36)	24 (21–34)	
Sex, in *N* (%)			
Female	1 (4)	5 (24)	
Male	25 (96)	16 (76)	
Race, in *N* (%)			
Black	2 (8)	2 (10)	
White	24 (92)	19 (90)	
Injury Severity Score (ISS)^a^, in median (IQR)	41 (30–45)	36 (34–43)	
Sequential Organ Failure Assessment (SOFA) Score, in median (IQR)	7 (5–9)	7 (4–9)	
Marshall Head CT Class^b^, in *N* (%)			
Class II (diffuse injury)	16 (62)	14 (67)	
Class III (diffuse injury & swelling)	6 (23)	7 (33)	
Class IV (diffuse injury & shift)	3 (11)	0 (0)	
Class VI (non-evacuated lesion)	1 (4)	0 (0)	
Outcomes			
Ventilator-free days, in mean ± SD	18.0 (0.1–20.5)	16.2 (5.5–20.1)	0.88
ICU length of stay, days, in median (IQR)	11 (6–18)	15 (10–21)	0.14
Hospital length of stay, days, in median (IQR)	18 (7–25)	20 (14–31)	0.23
In-hospital mortality, in *N* (%)	7 (27)	4 (19)	0.73
CFS^c^ during treatment	9.5 (8.0–11.0)	8.0 (6.0–9.0)	0.01
Fentanyl dose total^d^ mcg, in mean ± SD	8015 ± 5686	7375 ± 5931	0.71
Propofol dose total^d^ mcg/kg, in mean ± SD	1314 ± 1068	1377 ± 1382	0.86
Rancho^e^ score at discharge	3.5 (2.0–5.0)	5.0 (3.8–5.0)	0.33
GOSE^f^ at 3 months, in *N* (%)			0.89
GOSE1	8 (33)	5 (26)	
GOSE2	3 (12)	2 (11)	
GOSE3	7 (29)	5 (26)	
GOSE4	3 (12)	3 (16)	
GOSE5	1 (4)	2 (11)	
GOSE6	1 (4)	2 (11)	
GOSE7	0 (0)	0 (0)	
GOSE8	1 (4)	0 (0)	
GOSE^f^ at 12 months, in *N* (%)			0.84
GOSE1	8 (31)	5 (25)	
GOSE2	1 (4)	1 (5)	
GOSE3	4 (15)	1 (5)	
GOSE4	3 (12)	3 (15)	
GOSE5	2 (8)	3 (15)	
GOSE6	5 (19)	3 (15)	
GOSE7	3 (12)	3 (15)	
GOSE8	0 (0)	1 (5)	
QOLIBRI^g^ at 3 months	69 (52–76)	69 (54–82)	0.75
QOLIBRI^g^ at 12 months	70 (61–76)	69 (65–76)	0.82
Trail Making Test^h^ at 3 months	57 (36–159)	100 (53–134)	0.43
Trail Making Test^h^ at 12 months	62 (51–103)	39 (35–65)	0.04

*ICU* intensive care unit, *SD* standard deviation, *CI* confidence interval

Statistical testing: Pearson test used for categorial outcomes; Wilcoxon test used for continuous outcomes

^a^Injury Severity Score, ISS, ranges from 1 to 75, major trauma (or polytrauma) is defined as the ISS being greater than 15

^b^Marshall Head CT Class, CT scan derived metric, that places patients into one of six categories (I to VI) of increasing severity on the basis of findings on non-contrast CT scan of the brain; Marshall Class I, no injury, and Marshall Class V, evacuated mass lesion were exclusions for enrollment

^c^CFS, paroxysmal sympathetic hyperactivity and clinical features scale (range 0–18, with mild 1–6, moderate 7–12, and severe 13+)

^d^Total dose received during hospitalization

^e^Rancho, Rancho Los Amigos Levels of Cognitive Functioning Scale (range 1–10, higher is higher functioning)

^f^GOSE, Extended Glasgow Outcome Scale (range 1–8, higher is better functional outcome)

^g^QOLIBRI, Quality of life after Brain Injury scale (range 0–100, higher is better quality of life)

^h^Trail Making Test Part B

### Protocol adherence

Compliance and protocol adherence were high (99%, 1854 of 1872 doses possible), and blinding was intact. Reasons for non-administration of the 18 doses were as follows: hemodynamic parameters not met (11 cases), nursing error (4), pharmacy error (1), and problems with enteral access for clonidine (1). Study drug was discontinued in one patient due to rapid neurologic decline with plans for no further escalation of care. Study drug was never held due to physician preference, physician error, lack of IV access, or the patient not being present in the ICU (e.g., receiving MRI). Patients received non-study *β*-blockers or *α*_2-_agonists during the study period in two instances outside of the ICU, where *β*-blockers were administered intraoperatively by anesthesia personnel.

### Safety evaluation

Neither cardiac complications nor other serious adverse events occurred. A single patient in the treatment arm had a temporary asymptomatic junctional bradyarrhythmia that required no intervention, and the study drug was continued. This event was noted, and the patient remained in the study. No patients were removed from the study due to safety concerns. Study drug was never held due to concerns over clinical care. Non-study propranolol or clonidine were never required by clinical personnel in the ICU during the treatment period.

### Primary and secondary outcomes

**Fig. 2:**
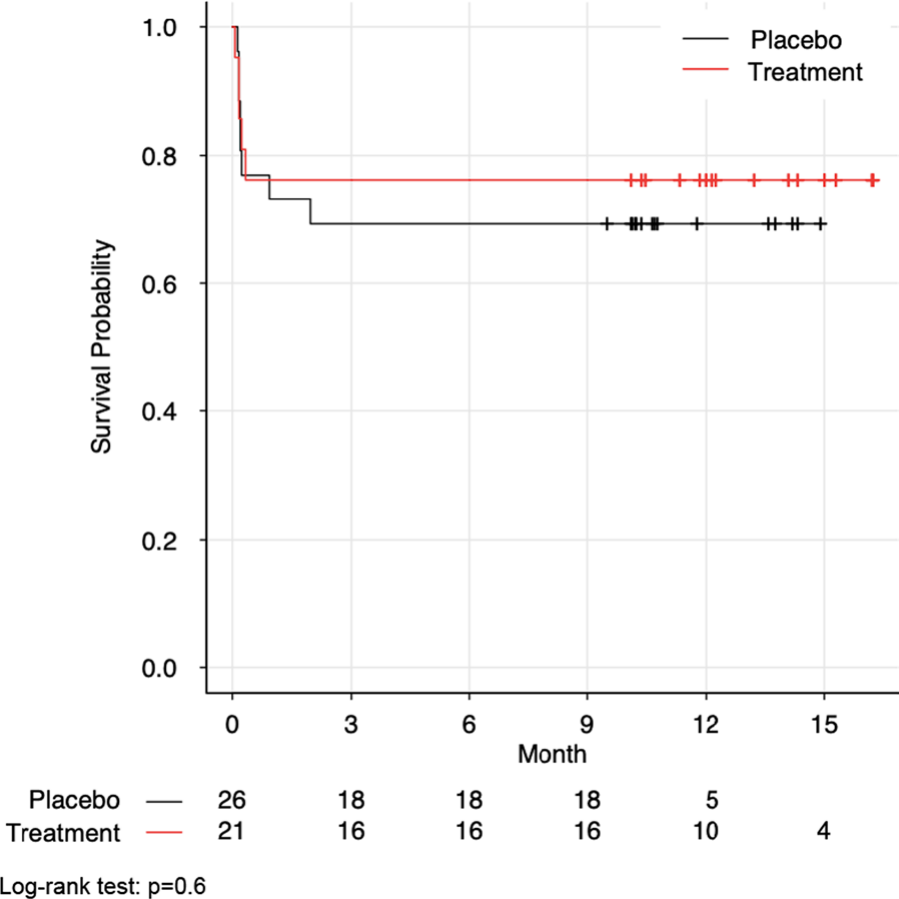
Kaplan–Meier survival curves of propranolol and clonidine (treatment) versus double placebo after severe traumatic brain injury

The mean ventilator-free days (VFD) did not differ between treatment and placebo groups (Treatment: 13.4 ± 9.2 vs. Placebo: 13.6 ± 9.9; mean difference 0.3 days, 95% CI − 5.4–5.8, *p* = 1.0). Similarly, we did not detect a significant difference in the secondary outcomes of agitation, LOS, in-hospital mortality (OR 1.4, 95% CI 0.3–6.7, *p* = 0.7), or improvements in long-term neuropsychological status despite > 90% follow-up (Table 1). Survival probability is shown in Fig. 2, which was similar between the treatment groups (*p* = 0.6). Sympathetic hyperactivity, as measured by the Clinical Features Scale (CFS), was significantly more severe in the placebo group (mean of 9.3 vs. 7.6, mean difference 1.7 points, 95% CI 0.4–2.9, *p* = 0.012). However, there was no significant difference in the secondary outcomes of plasma or urine catecholamine levels between pre-enrollment and study day 8 (Table 2).

**Table 2:** Pre-enrollment and study day 8 catecholamines after propranolol and clonidine (Treatment) versus double placebo after severe traumatic brain injury

	Placebo (*n* = 26) in median (IQR)	Propranolol and Clonidine (*n* = 21) in median (IQR)	*P* value
Enrollment catecholamines			
Plasma dopamine (pg/mL)	243 ± 775.6	64.7 ± 62.5	1
Plasma norepinephrine (pg/mL)	1007 ± 848	1232 ± 1082	0.3
Plasma epinephrine (pg/mL)	199.7 ± 265	177.7 ± 143.6	0.33
24 h urine dopamine (mcg/mL)	0.15 ± 0.11	0.12 ± 0.07	0.44
24 h urine norepinephrine (mcg/mL)	0.07 ± 0.06	0.06 ± 0.04	0.92
24 h urine epinephrine (mcg/mL)	0.02 ± 0.015	0.01 ± 0.01	0.5
Study Day 8 catecholamines:			
Plasma dopamine (pg/mL)	80.4 ± 98	56.4 ± 45.4	0.73
Plasma norepinephrine (pg/mL)	1082 ± 686	995 ± 724	0.84
Plasma epinephrine (pg/mL)	135.8 ± 98.3	93.8 ± 59	0.28
24 h urine dopamine (mcg/mL)	0.132 ± 0.07	0.16 ± 0.1	0.46
24 h urine norepinephrine (mcg/mL)	0.077 ± 0.04	0.1 ± 0.09	0.57
24 h urine epinephrine (mcg/mL)	0.014 ± 0.01	0.016 ± 0.01	0.89
Catecholamine difference (post–pre):			
Plasma dopamine (pg/mL)	27.8 ± 115.3	− 4.6 ± 64.1	0.5
Plasma norepinephrine (pg/mL)	92.1 ± 985.8	− 0.47 ± 939.2	0.61
Plasma epinephrine (pg/mL)	− 39.8 ± 246.3	− 82.9 ± 117.5	0.03
24 h urine dopamine (mcg/mL)	− 0.03 ± 0.14	0.04 ± 0.09	0.29
24 h urine norepinephrine (mcg/mL)	0.002 ± 0.08	0.04 ± 0.09	0.2
24 h urine epinephrine (mcg/mL)	− 0.003 ± 0.016	0.002 ± 0.02	0.65

## Discussion

This study is the only prospective double-blinded, placebo-controlled randomized trial of sympathetic blockade using propranolol and clonidine in patients with severe TBI. We found that it is safe and feasible to administer combined adrenergic blockade in these critically ill patients. However, we identified no significant differences in our primary outcome of ventilator-free days. The mean ventilator-free days were 13.6 in the placebo group and 13.4 in the intervention, whereas study drug administration only ran from up to 7 to 9 days (starting between 0 and 48 h after injury). This discrepancy may have resulted in a rebound effect of sympathetic hyperactivity that may have resulted in an inability to detect a difference. Although we did not identify significant differences in our primary clinical endpoint, we did find that despite this small sample, the treatment group receiving adrenergic blockade had improved scores on the Clinical Features Scale (CFS), a scale designed to assess symptoms of paroxysmal sympathetic hyperactivity (PSH), also known as “neurostorming”, “sympathetic storming”, and by other synonyms [31, 41–45]. Although findings regarding secondary outcomes such as this may have been due to failure to correct for multiplicity in our analysis, it is a signal to be further explored in future work, and also aligns with the treatment mentality of clinicians who use these pharmacologic interventions to decrease agitation after TBI.

In contrast to the lack of clinical benefit demonstrated in this study, several translational animal-model, retrospective, prospective and non-placebo-controlled trials support a link between adrenergic blockade with survival and even long-term cognitive benefit in severe TBI [21, 24, 46, 47]. The potential reasons for the conclusions of these non-randomized, non-blinded, and non-placebo-controlled trials are several. No studies to date have been able to effectively address potential unmeasured confounding of unknown or pre-existing cardiovascular disease in a diverse patient population, and thus, survival benefit of adrenergic blockade seen in these larger retrospective and prospective studies may be due to these unmeasured factors. In addition to excluding patients with known cardiovascular disease, we intentionally excluded patients > 65 years old to avoid confounding by undiagnosed cardiac risk factors that are prevalent in older patients with TBI, which may be the confounding mechanism by which other studies have found a survival benefit of adrenergic blockade.

Limitations of our study include small sample size and single-center design. Despite being the largest double-blinded placebo-controlled randomized trial to date of sympathetic blockade in patients with severe TBI, the power of the study was limited due to it being a small single-center pilot. Demographic and injury diversity was limited, with most patients being very young (< 30 years of age), male, white, and with enrollment criteria of severe TBI defined as GCS < 8. Marshall CT Class does not account for diffuse axonal injury (DAI), a characteristic seen on advanced neuroimaging, however, not usually typical in the acute phase of injury, and with an unclear relationship to the intervention delivered [48]. We used a critical care outcome, ventilator-free days (VFD), which may not best reflect the benefits of adrenergic blockade in these patients. However, we did measure secondary outcomes such as agitation and features of sympathetic hyperactivity using a validated clinical scale, which are common clinically cited reasons for these drugs. Mortality has been the primary outcome reported in the existing retrospective or observational studies; however, given the nature of this study as a pilot single-center double-blinded randomized placebo-controlled trial, it was not feasible to achieve the participant number needed to identify a statistically significant difference in mortality. Candidates for outcomes in future studies include mortality, severity of sympathetic hyperactivity, and ventilator-free days. Future trials may also utilize dexmedetomidine, with its more favorable pharmacokinetic profile and more widespread contemporary use in the ICU, in lieu of clonidine as an *α*_2_-agonist.

## Conclusions

It is feasible to conduct a rigorous double-blinded placebo-controlled trial of adrenergic blockade in young severe TBI patients without major adverse cardiovascular effects despite a complex ICU environment and drug administration schedule, while achieving long-term functional and neurocognitive follow-up. The practical execution of this single-center trial should support multi-center investigation powered for mortality and balanced for unmeasured confounders to determine whether the widespread practice of adrenergic blockade has any benefit after severe TBI.

## Data Availability

The datasets used and/or analyzed during the current study are available from the corresponding author on reasonable request.
